# Hospital and Institutional News

**Published:** 1916-01-29

**Authors:** 


### HOSPITAL AND INSTITUTIONAL NEWS.

### THE FLORENCE NIGHTINGALE MEMORIAL.

It may be within the remembrance of our readers that on the completion of the subscriptions required for this memorial (over £8,000 being forthcoming) we expressed a hope that arrangements should be made to place a replica of the statue of Florence Nightingale, with a suitable inscription, in Westminster Abbey, and also in St. Paul's Cathedral. Owing to the heavy contribution imposed in every case for its Restoration Fund, *i.e.* £200, the idea of a replica in Westminster Abbey has had to be abandoned. We understand, however, that Her Majesty the Queen has expressed a wish to unveil on some day in February the replica which the authorities of St. Paul's Cathedral have permitted the committee to place in the crypt. We are sure this news will be most welcome to the numerous readers of The Hospital who came forward with such marked generosity to make this memorial fund a success, and so prevented the failure with which it was once threatened.

### GRAVE ILLNESS OF MISS LÜCKES.

We very greatly regret to learn that Miss Eva C. E. Lückes is lying seriously ill at the London Hospital. We are glad to hear from the latest bulletins that she has rallied and is better. Her faithful nurses are encouraged, as we write, by a slight improvement. Miss Lückes has filled a considerable place in nursing affairs in this country for many years. She has devoted the best years of her life to the London Hospital, and the widest sympathy will be felt for her throughout the medical and nursing professions. The President of the London Hospital, Queen Alexandra, has paid more than one visit of sympathy and inquiry to the London Hospital during the past week. This is but one more instance of Her Majesty's invariable and tender sympathy for others. In this way Queen Alexandra has revealed herself to the nation, and won the universal affection which is felt for Her Majesty by all classes of the people throughout the Empire.

### TRURO'S NAVAL AUXILIARY HOSPITAL.

Some disappointment has been felt in Cornwall at the small number of wounded who have been sent there, fifty having been the largest number received at one time by the Royal Cornwall Infirmary. It was at one time proposed that a military hospital should be established at Truro, where the site and building of the workhouse offered advantages that were eventually turned to good account. With the support of the Guardians, and the consent of the Local Government Board, the inmates of the institution have been boarded out and, at the instance of the naval authorities, who appeared on the scene when the project for a military hospital had been abandoned, a naval auxiliary hospital has been established. The result of the alterations has been to provide fifteen wards, which, accommodating from eight to sixteen patients each, give a total number of 150 beds. The expense of the alterations has been met by the activity of the Cornish branch of the Red Cross Society, and the equipment of the wards has been provided by different areas in the county. The chief resident medical officer is to be a fleet surgeon, who will be assisted by a naval surgeon and, probably, certain medical men from Truro. Second-Lieutenant E. Beard, R.N.V.R., who has been acting as secretary of committee in charge of the arrangements, is to be quartermaster. There was little formal opening ceremony last week when the hospital was ready, but the "Cornwall County Royal Naval Auxiliary Hospital" is now an accomplished fact. A word of praise is due to Mr. Polwhele, the County Director of the Red Cross organisation, for the laborious part he has taken in carrying out the work.

### THE BRADFORD WAR HOSPITAL SCHEME.

It has been decided to provide a war hospital at Bradford which will give a total accommodation of 1,200 beds. In order that all the buildings may be grouped together on one site the whole of the buildings at Horton Green will be taken over, and the remaining Union patients will be moved elsewhere. The Lord Mayor of Bradford, pointing to what has been done at Leeds, has appealed for a sum of £25,000, so that the necessary additions may be carried out. The report of the committee responsible for the new scheme states that it is suggested that "after the war the new pavilions for patients be pulled down and the materials sold, the amount realised being returned to the Lord Mayor for the benefit of the new infirmary, or taken over by the Guardians, if found useful, at a valuation." The Lord Mayor also stated that he understood that the buildings could be ready within three months. The architect is Mr. Holland.

### MEDICAL ATTENDANCE ON SOLDIERS' DEPENDANTS.

When the war began the medical men of Guildford voluntarily placed their free services at the disposal of the dependants of those serving in the war; and the same benevolence was manifested in a good many other parts of the country by local groups of the profession. At a recent meeting of the Guildford Medical Ethical Society a resolution has been passed suspending this gratuitous attendance. The reasons for this change of policy are, it is stated, that the doctors find themselves hard pressed to get through their ordinary work, added to the services they are rendering free at the military hospitals; that their charity has in many cases been abused; and that many people are now claiming as a right what was offered as an act of kindness and consideration. Considering the extraordinarily generous provision made by the State for the dependants of soldiers, many of whom are far better off than ever in their lives before, it does seem unnecessary that the medical profession should shoulder this extra burden, save in the very exceptional cases. Possibly other district societies of the medical profession will follow Guildford's lead in this matter.

### THE RENOVATION OF THE ROYAL UNITED HOSPITAL, BATH.

This week sees the inauguration of a series of completed improvements which have been in process of achievement during the past two years at the Royal United Hospital, Bath. Prominent among those which the hospital's supporters have been invited to inspect are a new electrical department, a pathological laboratory, a new heating and ventilation system, an extended out-patient department, together with the repaving of the two main corridors with mosaic, and a good deal of inside painting. It will be recalled that the cost of these alterations has been met by the expenditure of two considerable legacies. The annual meeting has been fixed for January 31, three days after the date of the inspection, and it is hoped that the visitors, after seeing personally what has been done, will take a more lively interest than usual on this occasion. Annual meetings are as important as hospital organisers choose to make them, and to attract many people they must be made as interesting as, for instance, the new alterations are likely to prove.

### THE AMBULANCE DEPARTMENT OF THE ORDER OF ST. JOHN OF JERUSALEM.

The Earl of Plymouth has been promoted Sub-Prior of the Order of the Hospital of St. John of Jerusalem in England, and in his new capacity has, with the approval of the Grand Prior (the Duke of Connaught), appointed the Earl of Ranfurly, G.C.M.G., Knight of Justice of the Order, to be Director of the Ambulance Department, in the vacancy due to the Earl of Plymouth's advancement The latter was formerly chairman of the British Ophthalmic Hospital, Jerusalem, which is supported by the Order; he succeeded, if we remember aright, the Marquis of Breadalbane as Director of the Ambulance Department, and has a most honourable record, as everyone knows, of service to the common weal in other directions besides that of service to the sick. Lord Ranfurly has been for several years one of the selected members of the Council of the Order, and earned distinction as a colonial governor in the early years of the present century. Both these Earls are middle-aged men, as age is reckoned nowadays, and the Order may expect to have many years of valuable and energetic service from them.

### A MENTAL HOSPITAL SCHEME HELD UP.

The Government has ordered the L.C.C. to abandon work in connection with its eleventh mental hospital, which, at a total cost of half a million, is being erected at Epsom. At the Council on Tuesday it was declared that it is extravagant to suspend building operations, for in that case compensation is payable to contractors. Nevertheless, the Government require all available capital for war purposes, and obviously, if money is still spent in finishing the hospital, that mony cannot be used for the war. The need for the new hospital is great, but the need of the Government for money is greater, and so the building will not be completed till the war is over.

### THE HEALTH OF MUNITION-WORKERS.

The rather large Committee which was appointed by the Ministry of Munitions to report on various factors with respect to the output of munitions as affected by the health of the workers has already presented several reports; its latest memorandum is concerned with welfare supervision. The recommendations of the Committee are sensible enough, and their special interest from an economic and social aspect is that they apply equally to all kinds of factory work, whether in peace or war, and more especially to conditions peculiar to the present moment. The memorandum deals chiefly with the supervision required in factories where the workers are women and outlines a scheme of duties connected with "Welfare Supervision" which is certainly comprehensive. The qualities and qualifications laid down as desirable in a Welfare Supervision officer are numerous; the chief difficulty in the scheme that strikes the outside observer is the rarity of women possessing such talents and such experience. It is obvious from the powers and duties allotted to the officer that any failure to reach the highest standard of efficiency and character might lead to worse results than to have no supervision at all. Into the question of the salary advisable to attract such paragons as the memorandum contemplates the Committee do not enter. There is a tendency visible in these reports to be platitudinous instead of practical, of which this is an instance in point.

### THE ORGANISATION OF EX-PATIENTS' SUBSCRIPTIONS.

We have drawn attention before now to the organisation whereby the Batley Hospital secures an annual contribution from former patients, but as the plan is an unusual one it is perhaps worth while to say something of the local success by which it has been attended. There is a body known as the Hospital Ex-Patients' Committee, and this has given to the hospital funds a sum of £120 as a Christmas present. This sum, which forms a record, means that a total of £410 has been handed over to the hospital in four years, so that the ex-patients provide an annual income of about £100 to the hospital's funds. Every year the ex-patients' committee organises an entertainment, such as a whist drive and a dance, and the proceeds are the mainstay of the ex-patients' contribution. The idea is one which might well be considered for adoption in other places. In this connection it should be added that an extension scheme is in prospect by which three wards, containing seventy-eight beds, will be added to the hospital, first, that more wounded may be accommodated, and, secondly, that the hospital may be permanently enlarged, as has long been hoped for. Mr. A. W. Hanstock, the hospital's honorary architect, has prepared plans for new kitchens and wards, which will be built of brick. The cost is estimated at £5,000.

### MEDICINAL HERB FARMING.

The growing of medicinal herbs for profit is an industry which has been greatly neglected, both in this country and abroad. Manufacturers who have to import the raw material of various well-known drugs have often to complain of the variability in quality of such herbs, partly due to careless growing, gathering, storage, and the like. Some wholesale drug firms have found it advisable to start their own farms to overcome these drawbacks, with very considerable advantage to themselves and their customers. We note that this question is attracting interest in other circles; for it is announced that there is to be an organisation called the Herb-growing Association, affiliated to the Women's Farm and Garden Union. Members will be entitled to advice on herb-growing and the preparation and disposal of herbs. By co-operation the growing of different herbs will be regulated, so that the market may not be overstocked with some and be short of others. At present the growing of medicinal herbs is an unprofitable industry for the small grower because of the difficulty of disposing of his crops; but we understand that the new association intends to establish an arrangement for collection which should minimise the cost of transport and bring sellers and buyers in contact. The secretary's address is 45 Queen Anne's Chambers, London.

### THE SELLING OF DILUTED MILK.

A decision of great importance to milk vendors was given last week by the Lord Chief Justice. The case was a special one, stated by the County Justices of Rochdale, and raised the question as to the effect of a notice relating to the quality of milk sold. The vendor had been convicted and fined for selling milk which had admittedly been diluted. On his can was a notice to this effect, with the words "No standard guaranteed." The analyst's certificate showed that water had been added and fat abstracted. It was contended for the defence that there was no evidence of the abstraction of fat: the mere addition of water would reduce the percentage of fat. In point of fact, before the addition of the water the amount of fat present in the milk exceeded the standard required by the regulations. It was held that the certificate appeared to mean that in consequence of the addition of water there was a deficiency of fat, and that this was the whole point. The conviction was therefore quashed. It thus appears that if milk vendors choose they can sell diluted milk if it is made clear by notice that it is sold as such, a point of some importance in poor districts or where the milk supply is short.

### A NEW INSTITUTION AT DROITWICH.

The board of delegates of the Birmingham Hospital Saturday Fund has approved a scheme for establishing at Droitwich a home for the treatment of rheumatic cases. This course has been decided on owing to the closing of the two institutions to which rheumatic patients had formerly been sent, whereby as a temporary measure rooms in cottages in Droitwich had had to be taken. This plan not having proved entirely satisfactory, the project of the committee establishing its own home, which had been in the background for some time, presented itself. A place known as the Highfield property, which had formerly been built by a local doctor for the treatment of rheumatic patients, was available at a probable cost of £5,000. Twenty-five patients could be accommodated in it. A special collection of one penny a week for a limited period has been started by numerous firms, and in this way it is hoped that the purchase money may be quickly forthcoming. This will be a very important addition to the Birmingham Hospital Saturday Homes already in existence.

### SERIOUS ROBBERY AT A LONDON HOSPITAL.

Pat upon Lord Knutsford's letter in our last issue concerning robberies at hospitals comes the report in the public Press of the prosecution of a clerk, Alfred William Pearce, for extensive frauds on the London Homœopathic Hospital. It appears that Pearce embezzled in 1913 twenty-one sums of money aggregating £269; in 1914, forty-four sums (£418); and in 1915, sixty sums of a total of £797. We are indebted to the Chairman of the Homœopathic Hospital for a full account of the method pursued by this ingenious thief. It will be found in the Editor's Letter-Box, page 397.

### THE PAYMENT OF HOSPITAL CLERKS.

On being arrested, the clerk made a statement which is worth quoting, although it does not bear upon the method of fraud. "I have already made a clean breast of it," he is reported to have said to the sergeant. "I was going to make away with myself, but I could not get any veronal. The money has all gone in betting." The remark about veronal shows once more the need for proper restrictions on the sale of this drug, and, indeed, suggests that recent legislation is proving effective. In the second place the prisoner's confession that the money had "gone on betting" deserves notice. Betting may be simply a bad habit, and we have no means of knowing whether this unlucky young man was a victim to it or not. On the other hand no one will deny that betting may be encouraged by underpayment. We do not know what this clerk's salary was, but in view of the general admission that the secretariat of hospitals are poorly paid, this confession of betting serves to remind us that when the salaries of hospital clerks are being decided on, hospital managers should remember that unduly low salaries may occasion temptations to which no young man or woman ought to be exposed.

### A STRANGE AFFAIR AT CROYDON HOSPITAL.

A curious incident is reported in the *Croydon Guardian* as having come to light at an inquest upon a patient who died in the Croydon General Hospital. A labourer fell about nine feet from a scaffold to the ground. He was taken to the hospital in the car of a local practitioner, but was so little sensible of serious injury that he wanted to walk. Later in the day signs of internal abdominal mischief developed, and an operation was performed, which revealed a ruptured intestine; but, unfortunately, the man died. The strange part of the story is still to come. After the operation, at which the senior house surgeon was present, he packed his luggage and disappeared in the night, and so could not be subpœnaed for the inquest. The name of this individual and the explanation of his extraordinary conduct are not reported; probably the Coroner was satisfied as to the cause of death and would not bother himself about the vanished house surgeon. But the individual concerned owes it to the surgeons and the governors of the hospital to explain his act, and some means should be devised for calling him to account for it.

### A HOSPITAL SUNDAY SCHOOL.

A suggestion has been made by a correspondent of the *Church Family Newspaper* (January 21) which is worth careful consideration by chaplains and others in children's hospitals and in general institutions possessing children's wards. The idea is that of holding a Sunday school in hospital. It is stated that this is a practice at least in one children's hospital, one ward forming one class presided over by a well-trained and tactful teacher. It would, of course, be necessary to adapt methods to local conditions, but there seem some possibilities in the scheme suggested. Religious susceptibilities and antagonisms between different creeds are occasionally the most serious obstacles to such schemes.

### THE LAMBETH-SOUTHWARK DISPUTE.

On p. 341 of the issue of The Hospital for January 15 there appeared an account of some criticisms of the actions of the Lambeth Board of Guardians which had been expressed by the Southwark Guardians. It is pointed out to us that those criticisms, which were neither endorsed nor refuted in our columns, constitute an *ex parte* statement. The Lambeth Guardians do not admit the validity of the Southwark Board's arguments; and as their reply has been made public we must in fairness to them reproduce the substance of it. The Southwark Board, it may be remembered, passed on 250 paupers to the Lambeth Board in order to make room for soldiers in their own infirmary, undertaking to pay for the alterations and extensions necessary at the Lambeth institution. On receiving a bill for about £6,000 they declined to pass it, on the ground of unwarrantable extravagance. The Lambeth authorities' contention is that estimates were duly submitted to the Southwark Board in November last and approved by them; and that the work has actually been done within the estimated cost. They further point out in their own justification that the War Office have now voluntarily paid the bills in question; and this is certainly a strongly presumptive point against the alleged extravagant nature of the expenditure. This action by the War Office ends the dispute, which should, in our opinion, never have been allowed to reach the dimensions it has attained to.

### MILITARY HOSPITAL EXTENSIONS IN LEICESTER.

In a recent issue we referred at length to many excellent features of the 5th Northern General Hospital at Leicester, and especially to the series of open-air wards added to the original hospital under the supervision of Messrs. Everard, Son and Pick (architects). For some time past further extension of the hospital has received the consideration of the military authorities, and instructions have now been received for the provision of 520 additional beds, bringing the total number of beds in this hospital to 1,650. Some surprise has been caused locally that the Army authorities in this instance are carrying out the work. Doubtless the general scarcity of labour has been a consideration in arriving at this decision, but it should be remembered that the last extension was carried out in record time by the Leicester builders, who earned a handsome bonus on their achievement. The additions now approved will bring the number of military hospital beds in Leicester to 2,150, and with the auxiliary institutions 3,000 beds will be under the control of the 5th Northern General Hospital.

### A CAVELL COT AT STOCKPORT INFIRMARY.

An Edith Cavell Memorial Cot, endowed by Mr. Joshua Preston, J.P., has lately been formally presented to the Stockport Infirmary. The presentation ceremony was attended by a large gathering of supporters of the institution; and amongst those present was Miss Horn, one of Miss Cavell's assistants in Brussels, recently returned from Belgium. Lieut.-Col. A. J. Sykes, M.P. (treasurer), Mr. E. F. Ward (chairman of committee), Dr. Marriott (senior surgeon), and Mr. T. W. Potts (Mayor) spoke at the meeting; and a child was duly placed in the cot by the Mayoress. The Stockport Infirmary has allotted twenty-eight beds to soldiers, and is prepared with fourteen more when need arises. A new nurses' home has been built, and the programme of projected improvements includes a new out-patient and casualty department, the building of which has been already begun.

### ALLEGED NUISANCE AT A MILITARY HOSPITAL.

From time to time during the last two years references have been made in the columns of The Hospital to the nuisance arising at the Southwark Infirmary through the removal of refuse by the Camberwell Borough Council at their railway siding at East Dulwich. The Council now maintain that they take precautionary measures which tend to reduce the nuisance to a minimum, though the Guardians and their medical staff hold that despite the precautions the nuisance is not entirely removed. Now the medical officer in charge of the military hospital has written to the railway company complaining as to the offensive state of the siding, which it is alleged is caused by the way the Council handle the refuse there. The railway company have appealed to the Council, and the Public Health Committee have passed a resolution that they keep a continuous and close watch over the handling and clearing of all refuse sent in by them to the siding, and can find no reason for complaint, though they agreed that further supervision should be given by the railway company regarding the removal of manure and other refuse sent in by the general public. There is here an effort to throw the burden on the railway company, but it remains to be seen whether the military medical staff, having taken the matter up, will be satisfied with the non-action of the Camberwell Council. Previous efforts to get the Council to remedy the nuisance have proved futile, though the Guardians did everything in their power to compel Camberwell to carry out its duties under the Public Health Acts. In the interests of public health and decency it is to be hoped that the Camberwell Council will speedily be compelled by mandamus or otherwise to discharge the duty they have so long neglected.

### HONOURS FOR THE NURSING PROFESSION.

In dealing last week with the honours conferred on members of the nursing profession in recent *Gazettes* we omitted to add our congratulations to the authorities of the Leicester Royal Infirmary and the Lincoln County Hospital, whose matrons have been honoured by the grant to them of the Royal Red Cross (First Class). Leicester is one of the most interesting of our provincial voluntary hospitals, and had attained a high state of efficiency before the outbreak of the war. Lincoln County Hospital is excellently planned, and London is indebted to it for having sent a very knowledgeable and experienced matron to become the responsible and able head of the nursing and domestic departments of the new King's College Hospital.

### COVENTRY AND WARWICKSHIRE HOSPITAL.

We have the pleasure to call our readers' attention to the plans of the new wards and reconstruction of this progressive provincial hospital. Sir Henry Burdett published a report on this institution in 1909 (The Hospital, December 25, 1909, page 365). It is interesting to note that the architect, Mr. Frank L. Cox, was particularly anxious that the plans of the buildings as they are to-day should be published in The Hospital, because it was "greatly on account of Sir Henry "Burdett's remarks that the larger portion of the "extension scheme was carried out when it was. At "the date of Sir Henry's visit in 1909 only the "administrative portion of the scheme and the "laundry had been erected, and he expressed very "strong views as to the inadequate condition of the "remaining portion of the institution. The com-"mittee proceeded to consider plans of the largest "and most important portion of the scheme, namely "the wards, operation theatre, main kitchen, and so "forth." We feel a word of appreciative recognition is due to the committee of this institution, and to its chairmen, Mr. Alfred Herbert and subsequently Mr. Edward M. Iliffe, who have done splendid work that has proved most fruitful in results for good.

### THE BRITISH HOSPITAL AT PETROGRAD

The British Hospital for Russian wounded recently established at Petrograd has been visited by the Metropolitan of that city, who was received by the British Ambassador, Sir George Buchanan, Lady Georgina Buchanan, and the Rev. Bousfield Lombard, the British chaplain. Sir George's daughter, Miss Muriel Buchanan, is one of the nurses. The Metropolitan delivered a sympathetic address, full of good wishes for the Allies and of prayer for the triumph of their cause. Incidentally the telegram which describes the honour thus paid to the British Hospital acquaints us with the fact that for some time "Rule Britannia" has replaced "God Save the King" in Russia as the complimentary tune for the British: this was owing to the similarity between the latter and the German national anthem. Now, however, by desire of King George, "Rule Britannia" is to be abandoned in favour of the more correct anthem: doubtless the sheer impossibility of anyone in Russia desiring to play or to hear the German tune will prevent any future chance of misconceptions over this small point.

### THE COLLEGE OF NURSING.

Judging from the number and quality of the letters we have received during the past week from matrons, sisters, and nurses who are desirous of expressing their interest in the immediate establishment of a College of Nursing as outlined in our issue of January 15, page 307, the profession as a whole are alive to the importance of this proposal and the value of their united co-operation at the present juncture. From all parts of the country and from all ranks of the profession requests have reached the *Nursing Mirror* to include the writer's name as favourable to the College of Nursing. The matron of an important London Poor-Law Infirmary writes: "I very gladly welcome "the proposition of a College of Nursing . . . and "sincerely hope that those who are so kindly in-"teresting themselves in this project will have all "the support and co-operation they require to bring "this idea to a successful issue." We shall be glad to receive the names and addresses of all matrons, sisters, and nurses, with particulars of their training, who are interested in the establishment of this College. Letters may be addressed to the Editor of The Hospital, 28 and 29 Southampton Street, Strand, London, W.C.

### THE "SOUTHERN CROSS."

We have to thank the Editor for sending to us the first number of the "*Southern Cross*," the journal of the 1st Southern General Hospital, R.A.M.C. (T.), Birmingham. In a foreword, Lieutenant-Colonel F. Marsh informs us that the title of the magazine is a crasis of the designation of the hospital and the emblem of its work. It is certainly a happy title, and covers a series of articles, verses, and illustrations which suggest a popular future for its pages. Perhaps its best feature is a drawing, signed "Gene," which, representing a flamingo disguised as an officer, forms an admirable cartoon, under the ingenious title "Who's Zoo, No. 1." Another good illustration is "Two O'clock Parade" (unsigned). The articles, enlivened with the usual sprinkling of jokes and stories in which the orderly comes in for some good-humoured chaff, include an account of the work performed by the Birmingham Volunteer Motor Transport in meeting the convoys of wounded, and conveying them from the station to the hospital; a description of the origin of the R.A.M.C. badge; accounts of the hockey and football matches which have been played; and a description of the numerous entertainments that have been given. It should be added that the "1st Southern" includes five sections, so there should be a wide area from which contributors and incidents of interest can be drawn. The price of the magazine is 4d.

### A HOSPITAL FOR NEURASTHENIC SOLDIERS.

The new military hospital for neurasthenic soldiers is reported to be ready for occupation. Taken over by the War Office from the London County Council, the Maudsley Hospital, to give it its original name, is being provided with 150 beds, or fifty more than it was originally planned to accommodate. When the Board of Control several months ago mentioned the wish of the Army Council to obtain the use of the Maudsley Hospital for soldiers suffering from nervous disorders, the War Office undertook to refund any cost that might be incurred at a later date when the time comes for it to revert to its original purpose. The hospital is to be attached to the 4th London General Hospital (King's College), to which it is contiguous.

### DISPENSARY FINANCES IN WAR-TIME.

An interesting sidelight on the financial situation of dispensaries during the war was revealed at the annual meeting of the Bristol Dispensary, when Mr. Fenwick Richards explained how it came about that the subscriptions had fallen by £100. Some of the large firms, he said, were giving smaller subscriptions, and he did not believe that half a dozen new subscribers had joined during the past twelve months. The circumstance, however, arising most directly out of the war which had affected them was the prosperity of the wives of men who had gone to the Front. These, he said, were generally well looked after, and in some cases were in receipt of more money than their husband's former wages. The result was that some of these women, instead of seeking dispensary notes, called in on their own account medical men in the districts where they lived. This is interesting not only as showing the new situation which the dispensaries have to face, but also because it proves the independent spirit of the average patient, who, at bottom, always favours complete freedom of choice in the matter of professional attendance, and is averse to any system, let alone the opposite extreme of the panel, which limits this in any way. It is fair to add that, notwithstanding reduced subscriptions, the dispensary still possesses a small balance on the right side.

### AN INTERNED DOCTOR'S TUBERCULOSIS SERUM

In a somewhat sensational fashion attention has been drawn in the lay Press to the German scientist Dr. Frederic Mehnarto, who has for some time been interned in the Alexandra Palace. He is said to have been allowed to work in his laboratory under supervision, and to treat an eminent person with the special serum associated with his name. This serum, which was put on the market under the name Contra-toxin No. 4, has been for nearly three years under trial, and was extensively used in the treatment of tuberculosis, especially at the hospital in Margaret Street, near Oxford Circus. The serum was intended to fulfil the principle of passive immunisation in contradistinction to that of active immunisation which governs the employment of most of the tuberculins commonly used. Dr. Mehnarto's contra-toxin stands more or less in the same category as any anti-toxin, such as that used for diphtheria. It is said to be prepared from the serum of actively immunised, cold-blooded animals which are resistant to invasion by the tubercle bacillus. Its object when injected into human sufferers from tuberculosis would be to neutralise the poison of the tubercle bacilli and to confer a temporary immunity. Similar anti-serums have been tried from time to time without definite results, but Dr. Mehnarto's contra-toxin has apparently met with some degree of success. It must, however, be distinctly regarded as still on its trial.

### THIS WEEK'S DRUG MARKET.

With very few exceptions the values of drugs are firmly maintained, and in some cases the tendency continues in an upward direction. One of the exceptions is ipecacuanha, which, in consequence of the arrival of further supplies, is somewhat cheaper, but any further substantial decrease in value is hardly to be expected in view of the large demand for emetine for the treatment of dysentery. As was anticipated, the price of cocaine has again advanced materially, and a considerable business has been done at the higher figure; there appears to be no reason to expect that the shortage of supplies is more than temporary, and, while it might be advisable for hospitals to cover their immediate requirements, it would perhaps be unwise to do more than this, as the price now ruling may not be maintained for very long. Potassium permanganate is again dearer. It seems probable that there will be a further advance in the value of bromides, and makers are only able to supply in limited quantities. The high prices of acetanilide, acetyl-salicylic acid, salicylate of soda, phenacetin, antipyrine, and in fact of practically all the synthetic drugs are firmly maintained, and no relief of the scarcity appears to be imminent. The Norwegian cod-fishing season will shortly commence, and in the meantime the market for cod-liver oil is quiet.

